# Genome-wide determination of poly(A) sites in *Medicago truncatula*: evolutionary conservation of alternative poly(A) site choice

**DOI:** 10.1186/1471-2164-15-615

**Published:** 2014-07-21

**Authors:** Xiaohui Wu, Bobby Gaffney, Arthur G Hunt, Qingshun Q Li

**Affiliations:** Department of Automation, Xiamen University, Xiamen, China; Department of Plant and Soil Sciences, University of Kentucky, Lexington, KY USA; Key Laboratory of the Ministry of Education on Costal Wetland Ecosystems, College of the Environment and Ecology, Xiamen University, Xiamen, China; Department of Biology, Miami University, Oxford, OH USA; Rice Research Institute, Fujian Academy of Agricultural Sciences, Fujian, China

**Keywords:** Alternative polyadenylation, RNA processing, Antisense, Evolutionary conservation, Legume, *Medicago truncatula*

## Abstract

**Background:**

Alternative polyadenylation (APA) plays an important role in the post-transcriptional regulation of gene expression. Little is known about how APA sites may evolve in homologous genes in different plant species. To this end, comparative studies of APA sites in different organisms are needed. In this study, a collection of poly(A) sites in *Medicago truncatula*, a model system for legume plants, has been generated and compared with APA sites in *Arabidopsis thaliana*.

**Results:**

The poly(A) tags from a deep-sequencing protocol were mapped to the annotated *M. truncatula* genome, and the identified poly(A) sites used to update the annotations of 14,203 genes. The results show that 64% of *M. truncatula* genes possess more than one poly(A) site, comparable to the percentages reported for *Arabidopsis* and rice. In addition, the poly(A) signals associated with *M. truncatula* genes were similar to those seen in *Arabidopsis* and other plants. The 3′-UTR lengths are correlated in pairs of orthologous genes between *M. truncatula* and *Arabidopsis*. Very little conservation of intronic poly(A) sites was found between *Arabidopsis* and *M. truncatula*, which suggests that such sites are likely to be species-specific in plants. In contrast, there is a greater conservation of CDS-localized poly(A) sites in these two species. A sizeable number of *M. truncatula* antisense poly(A) sites were found. A high percentage of the associated target genes possess *Arabidopsis* orthologs that are also associated with antisense sites. This is suggestive of important roles for antisense regulation of these target genes.

**Conclusions:**

Our results reveal some distinct patterns of sense and antisense poly(A) sites in *Arabidopsis* and *M. truncatula*. In so doing, this study lends insight into general evolutionary trends of alternative polyadenylation in plants.

**Electronic supplementary material:**

The online version of this article (doi:10.1186/1471-2164-15-615) contains supplementary material, which is available to authorized users.

## Background

Polyadenylation is the cleavage in a specific location of the 3′-end of pre-mRNA and the addition of a poly(A) tail to form a mature mRNA. Polyadenylation is a key process during eukaryotic gene expression, playing an important role in mRNA stability, translation and transport [1]. If a gene possesses more than one poly(A) site, then it undergoes alternative polyadenylation (APA). APA leads to the formation of mature mRNAs with different natures. Thus, the selection of poly(A) sites located in protein coding regions or introns may result in different protein products. Even within 3′-UTR, different APA sites may regulate mRNA stability and translatability by altering the ability of the mRNA to be regulated by RNA-binding proteins or microRNAs.

Recent studies have shown extensive networks of potential APA in different species and have linked APA to epigenetic regulation and many biological processes [1, 2]. As many as 33% of the 4057 genes in *Chlamydomonas reinhardtii* have at least two unique poly(A) sites [3]. In higher plants (*Arabidopsis* and rice), more than 70% of expressed genes possess more than one poly(A) site [4–7]. In animals, APA affects transcripts from 55% of zebrafish genes [8], 43% of genes are annotated with more than one 3′-UTR isoform in *Caenorhabditis elegans* [9], and almost 70% of known human genes have multiple poly(A) sites [10].

Many studies have shown the evolution patterns of APA in various organisms, especially mammals. Based on 3′-ESTs with poly(A) tails, Yan et al. [11] found four distinct classes of patterns of APA in the human, mouse, and rat genomes: tandem poly(A) sites, composite exons, hidden exons, and truncated exons. Also using ESTs, Ara et al. [12] studied the poly(A) site evolution in mammalian genes in humans and mouse and identified about 4800 conserved poly(A) sites. Galante et al. [13] generated a catalog of conserved sense-antisense pairs occurring in the human and mouse genomes using ESTs and massively parallel signature sequencing data and suggested that these might be involved in several cellular phenomena. Lee et al. [14] established a conserved pattern for APA in several vertebrate species, and found that the 3′-ends of mRNAs could be dynamically modified by transposable elements through evolution. Derti et al. [10] applied a polyA-seq method for high-throughput sequencing of 3′-ends of polyadenylated transcripts to identify genome-wide poly(A) sites in the human, rhesus, dog, mouse, and rat genomes.

All of the above computational analyses mainly focused on the conservation study of APA in mammals. The evolution patterns of polyadenylation in plants are still largely unknown. *Medicago truncatula* is a model plant for the study of legume biology, the draft sequence of which has been completed [15]. However, the annotation of the genome of *M. truncatula* remains relatively incomplete, especially when it comes to transcript models. Specifically, there is no collection of poly(A) sites available in *M. truncatula*. Consequently, the majority of the 3′-UTRs are not fully annotated [16]. Here, using a high-throughput sequencing protocol, we generated a comprehensive and high-resolution map of poly(A) sites utilized in leaf and root tissues of *M. truncatula*. Our results show that poly(A) signals in *M. truncatula* are similar to those seen in other plants, and that the scope of possible APA (reflected in the number of *M. truncatula* genes possessing more than one site) is also similar to that reported for other plants. In addition, our results reveal a rather low extent of evolutionary conservation of APA involving intronic poly(A) sites, and greater conservation of sites situated within protein-coding regions, as well as sites associated with antisense transcripts.

## Results

### High throughput determination of poly(A) sites in *M. truncatula*

To determine poly(A) sites encoded in the *M. truncatula* genome, the poly(A)-tag-seq (PAT-seq) approach described by Wu et al. [7] was used. This entails the generation of Illumina-compatible short cDNA tags that include the mRNA-poly(A) junction. For this, cDNA was synthesized with RNA isolated from pooled leaves and roots from *M. truncatula* plants. Reverse transcription reactions used a primer containing (from the 5′ to 3′ direction) sequences compatible with the Illumina high throughput sequencing process, a sample-specific bar code, an oligo-dT tract, and a 3′-terminal two nt anchor to promote priming at the 3′UTR-poly(A) junction. Reverse transcription reactions also included a so-called SMART adapter intended to promote reverse transcriptase-mediated template switching at the 3′ ends of completed cDNAs [17]. The template switching serves to “add” two restriction enzyme sites and a suitable sequence for subsequent PCR amplification of the resulting cDNAs. Subsequently, the cDNA was amplified, digested with one of two restriction enzymes, and the appropriate Illumina-based sequencing adapter appended to the digested molecules by ligation. Tags so prepared were recovered, amplified, and submitted for paired-end sequencing on an Illumina GAIIx instrument.

Approximately 17.9 million paired-end sequences were generated using this approach. These sequences were processed so as to identify high-confidence tags; this processing included an initial mapping of the 3′ end (oligo-dT/dA-containing) tags to the current release of the *M. truncatula* genome using stringent match parameters, followed by a step that removed 3′ end tags that did not have a corresponding mapped 5′ paired end tag. This process yielded approximately 2.7 million high-confidence tags (Table 1). The low yield reflects the stringency of the initial mapping, the difficulty of sequencing the 18 nt oligo-dT/dA tract present in each 3′ end tag, and the frequency with which PATs mapped to multiple genomic positions; these latter tags were segregated for separate analysis (see Additional file 1: Table S1). Finally, tags that mapped to oligo-A tracts of 6 or more bases in genomic locations were discarded, so as to remove possible internal priming artifacts. This yielded more than 2 million high-quality tags for further analysis.

**Table 1:** **Statistics of the sequencing data from** *M. truncatula*

Processing stage	Tag#
Raw tags	17,850,997
Poly(T) tags^1^	14,164,786
Mapped Poly(T) tags^2^	4,972,758
Paired poly(T) tags^3^ (internal priming excluded)	2,714,942

^1^The tags that started with a stretch of Ts.

^2^The tags that started with a stretch of Ts and could be mapped uniquely to the genome.

^3^The mapped Poly(T) tags whose paired-end partners could be mapped uniquely to the genome.

### Distribution of sense and antisense poly(A) sites

The curated poly(A) tags (PATs) were mapped to the latest annotation of the *M. truncatula* genome (release Mt4.0v1) and the positions of PATs within annotated regions determined. To do this, the *M. truncatula* genome annotation was modified so as to better estimate the occurrence of poly(A) sites within putative 3′-UTRs; this was done because the current annotation is largely based on protein-coding predictions and thus likely to lack 3′-UTR information. Thus, genes that had no sequences downstream from the end of the protein-coding region were extended by 400 bp; this value reflects the typical average length of 3′-UTRs in other plant species (289 nts in rice; [4]) and the observation that an extension of annotated 3′-UTRs by 120 bp improves the representation of poly(A) sites in the *Arabidopsis* genome [7]. *M. truncatula* genes that had some possible 3′-UTR sequence were extended by 200 bp, again to improve the “recovery” of PATs that fall within authentic 3′-UTRs. Subsequently, PATs that clustered together (separated by fewer than 24 nts) were grouped into so-called Poly(A) site Clusters, or PACs [7] to reduce the impact of microheterogeneity on subsequent analyses.

**Figure 1:**
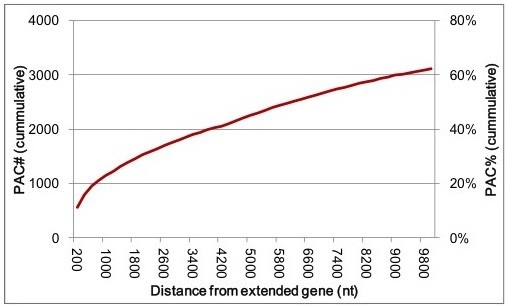
**Distances of intergenic PACs from neighboring genes.** *M. truncatula* genes were extended at their 3′ ends as described in Methods, and the distances between the ends of the extended genes and intergenic PACs (those that fall between extended annotations) were plotted. The cumulative sum of all intergenic PACs is plotted on the y-axis. Note the non-canonical scale for the x-axis.

The 2 million high-confidence PATs define approximately 42,600 sense-oriented PACs (Table 2). The majority (63%) of these fall within extended 3′-UTRs, while 6% and 10% map to introns and protein-coding regions, respectively (Table 2). Few PACs mapped to 5′-UTRs. 7.3% of the PACs fell within ambiguous regions of the annotated genome (e.g., regions that, owing to alternative transcription or RNA processing, may fall within UTRs or coding regions), and 11% mapped to putative intergenic regions. Of the 4843 PACs that mapped to intergenic regions, 76% fell farther than 1000 bp from the end of the nearest protein-coding region (Figure 1), suggestive of the existence of a number of unannotated transcription units. The 37,024 PACs that mapped to annotated regions fell within 14,203 genes. Of these 14,203 genes, 9077 (64%) possessed more than one PAC (Table 3).

**Table 2:** **Genomic distribution of the sense PACs in** *M. truncatula* **and** *Arabidopsis*

Region	*M. truncatula*				*Arabidopsis*			
	PAC#	PAC%	PAT#	PAT%	PAC#	PAC%	PAT#	PAT%
3′-UTR^1^	26915	63.2	1724860	84.20	34189	64.2	2193391	94.0
5′-UTR	34	0.08	142	0.01	185	0.35	6431	0.28
AMB^2^	3104	7.29	179244	8.75	2532	4.75	83859	3.59
CDS	4470	10.5	29468	1.44	3759	7.05	10899	0.47
Intergenic	4843	11.3	69888	3.41	7233	13.6	21142	0.91
Promoter^3^	724	1.70	5130	0.25	2994	5.62	10000	0.43
Intron	2501	5.87	39787	1.94	2296	4.31	6711	0.29
Pseudogenic_exon^4^	-	-	-	-	101	0.19	1192	0.05
Exon^4^	-	-	-	-	398	0.75	2771	0.12
Total	42591	100	2048519	100	53289	100	2333625	100

^1^The 3′-UTR region is the extended region as defined in the text.

^2^AMB: Ambiguously mapped at regions that have different annotations due to alternative transcription or RNA processing.

^3^The promoter region is defined as the region 2000 upstream from the 5′-UTR.

^4^In the annotation file of *M. truncatula*, there are no pseudogenic_exon and exon annotations.

**Table 3:** **Number of** *M. truncatula* **genes with different number of PACs**

PAC# ^1^	Gene# ^2^	Gene% ^3^
1	5126	36
2	3386	24
3	2282	16
4	1446	10
> = 5	1963	14
Total	14203	100

^1^Numbers of PACs possessed by a gene.

^2^Numbers of genes possessing the number of PACs indicated in the first column.

^3^Percent of all *M. truncatula* genes that possess the number of PACs indicated in the first column.

To assess the reliability of these PACs, publically available ESTs were used. Only 2% (5529 of 259,740) of the *M. truncatula* ESTs in the public collection have a poly(A) tail, thus defining 5529 poly(A) sites. A total of 4302 (78%) of these sites are within 50 nt of one or more PACs that are defined by the PATs. Since there is extensive 3′ end heterogeneity in plant transcription units, and given that the EST-derived poly(A) sites define a single site for each corresponding gene, this spatial correspondence between ESTs and PACs indicate that PACs effectively recover the 3′ ends of cloned cDNAs.

To gauge the similarity with *Arabidopsis*, the previously described PAT collection [7, 18] was re-analyzed using the same criteria as were used to map the *M. truncatula* tags. As shown in Table 2, the genomic distributions of sense-oriented PATs and PACs were similar in the two organisms. 10% more PATs mapped to 3′-UTRs in *Arabidopsis* than in *M. truncatula*; however, this difference could be traced to a larger number of PACs in *M. truncatula* that mapped to ambiguous regions and to intergenic regions. This difference probably reflects the state of annotations of the two genomes.

**Figure 2:**
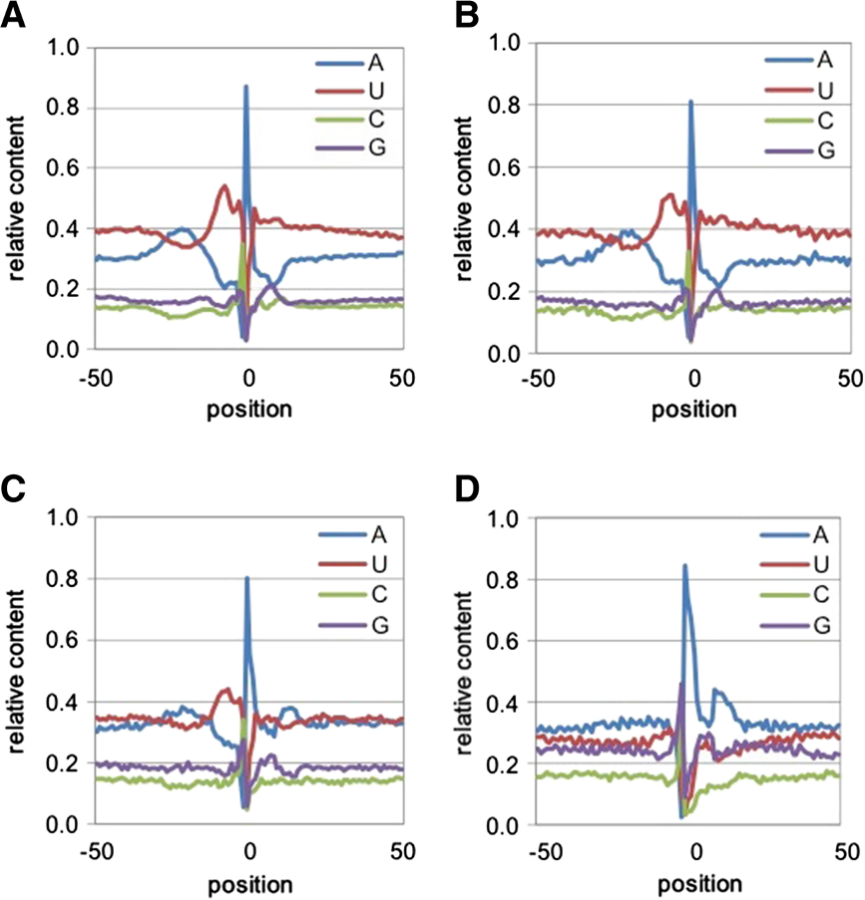
**Nucleotide compositions of the sequences surrounding** *M. truncatula* **PACs with different genomic regions.** Position-by-position base composition of PACs that map to 3′-UTRs **(A)**, introns **(B)**, intergenic regions **(C)**, protein coding regions **(D)**. Y-axis values are the fractional nucleotide content at each position (plotted along the x-axis); individual traces are color coded as indicated. On the x-axis, “0” denotes the actual cleavage/polyadenylation site.

A plot of the frequencies of the four nucleotides as a function of position with respect to the poly(A) site is a useful tool for assessing PAC and PAT quality; accurate demarcation of poly(A) sites is expected to yield defined poly(A) signals, while random localizations of mapped PATs is expected to yield a uniform, unbiased nucleotide distribution in the regions of PACs. When this is done for PACs that fall within the general genomic regions (3′-UTR, protein-coding regions, introns, and intergenic regions), the profiles shown in Figure 2 are obtained. The profiles for PACs that fall within 3′-UTRs and introns have similar patterns – a relatively high U content between 30 and 100 nts upstream from the poly(A) site, a distinctive A-rich region centered around -20 nt, and a cleavage site that consists of a YA dinucleotide embedded within a very U-rich region (Figure 2A and B). A similar result is seen for sites that fall outside of annotated regions (Figure 2C; except a slight less difference seen between A and U content); this implies that these sites are authentic and analogous to sites that fall within 3′-UTRs and/or introns. In contrast, for CDS-localized PACs (Figure 2D), the defining feature is that these are embedded within regions of elevated A + G content. These profiles are decidedly non-random, and they mirror results obtained in *Arabidopsis* [7].

A large percentage of the PACs defined in these analyses are derived from single PATs (Additional file 1: Table S2). For all four classes of sites (3′-UTR, intronic, CDS, and intergenic), the single-nucleotide profiles associated with these sites (Additional file 2: Figure S1) are similar to the profiles obtained when studying all PACs (Figure 2) as well as the profiles obtained when studying PACs defined by more than one PAT (Additional file 2: Figure S2). These results indicate that PACs defined by single PATs are authentic poly(A) sites.

Using this information, the *M. truncatula* genome annotation was updated to reflect the new information regarding poly(A) sites (and corresponding 3′-UTRs); in so doing, the annotations of some 14,203 genes were updated (Additional file 3).

5531 PACs were identified that were oriented on the opposite strand (antisense) as the gene to which they mapped (Table 4). 2278 (41%) of these PACs could be attributed to transcription from an adjacent, oppositely-oriented gene (cases 1 and 2 in Table 4). In addition, 29% occurred in genes that had nearby genes from which convergent transcription was possible, even though the annotation does not support this possibility. The remaining 31% of antisense PACs could not be attributed to nearby genes.

**Figure 3:**
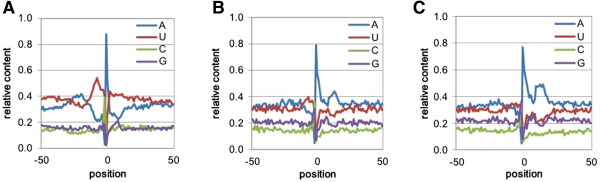
**Nucleotide compositions of the sequences surrounding** *M. truncatula* **antisense PACs with different genomic regions.** Position-by-position base composition of PACs that map to overlapping genes **(A)**, nearby genes **(B)**, of the orphan antisense PACs **(C)**. Y-axis values are the fractional nucleotide content at each position (plotted along the x-axis); individual traces are color coded as indicated. On the x-axis, “0” denotes the actual cleavage/polyadenylation site.

To confirm that these antisense PACs were authentic poly(A) sites, and not the results of random or spurious mappings, the nucleotide compositions of regions surrounding these sites were determined. Sites that originated from overlapping transcription bore profiles that were similar to sense-oriented sites located in 3′-UTRs, introns, and intergenic regions (compare Figure 3A with Figures 2A-C). In contrast, antisense sites that may be associated with transcription from nearby genes (Figure 3B) or orphan antisense PACs (Figure 3C) were more similar to sense-oriented sites that fall within protein-coding regions (Figure 2D); especially apparent are the pronounced elevated A contents and reduced U contents downstream from these different classes of sites.

**Table 4:** **Distribution of antisense PACs in** *M. truncatula* **and** *Arabidopsis*

Case ^1^	*M. truncatula*				*Arabidopsis*			
	PAC#	PAT#	PAC%	Gene pair#	PAC#	PAT#	PAC%	Gene pair#
1	1743	99321	30	1014	9045	780712	49	5325
2	535	41069	9	282	2059	67714	11	1288
3	1562	30389	27	-	4808	12220	26	-
4	1661	7279	29	-	2552	6064	14	-
Total	5531	177758	100	1296	18464	863710	100	6613

^1^Cases 1–4 are as described in Wu et al. [7]. Specifically:

Case 1 – antisense PACs map to the 3′-UTR of an adjacent, convergently-transcribed gene.

Case 2 – antisense PACs map to the CDS or 5′-UTR of an adjacent, convergently-transcribed gene.

Case 3 – antisense PACs are near the end of an adjacent, convergently-transcribed gene, but do not map to the nearby annotated gene.

Case 4 – antisense PACs cannot be attributed to an adjacent, convergently-transcribed gene.

In *Arabidopsis*, many more antisense PACs (49% of the total) could be attributed to overlapping genes than was seen in *M. truncatula* (32%; Table 4). In addition, many fewer “orphan” antisense PACs that could not be attributed to nearby genes were seen in *Arabidopsis* (14%) than in *M. truncatula* (31%; Table 4). More generally, there were only 30% as many antisense PACs in *M. truncatula* as in *Arabidopsis*. In contrast, the numbers of sense PACs identified in *M. truncatula* were 80% of those found in *Arabidopsis* (Table 2).

### Evolutionary comparisons of poly(A) sites

**Figure 4:**
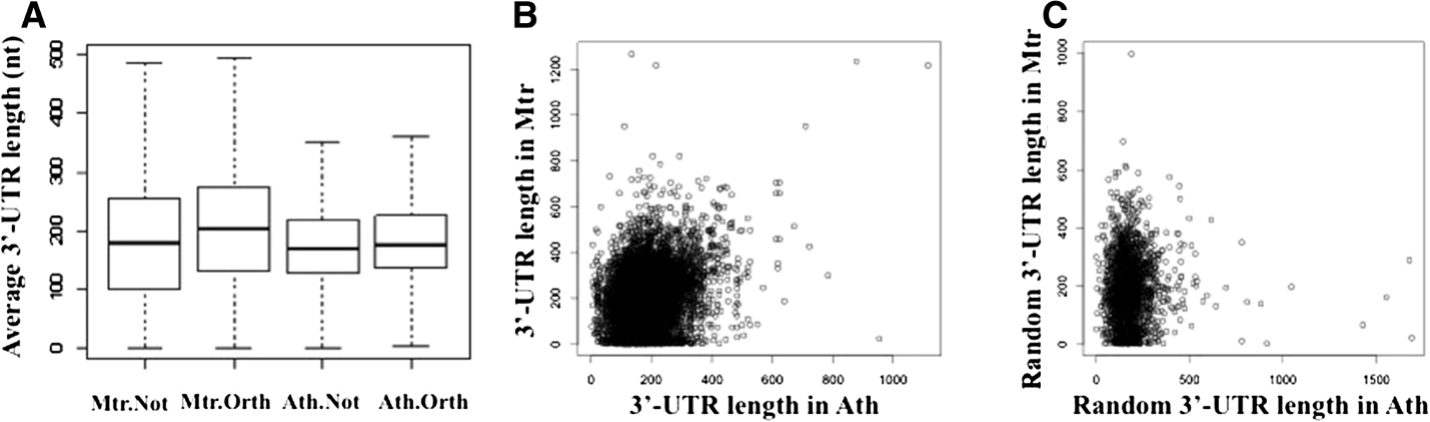
**3′-UTR lengths in** ***Arabidopsis*** **and** ***M. truncatula*** **genes. A**. The average 3′-UTR length from non-orthologous genes in *M. truncatula* (Mtr.Not), orthologous genes in *M. truncatula* (Mtr.Orth), non-orthologous genes in *Arabidopsis* (Ath.Not), and orthologous genes in *Arabidopsis* (Ath.Orth). Numbers of genes are: 6388, 8876, 7060, and 8876, respectively. Median values (nt) are: 180, 203, 169, 176, respectively. The Wilcoxon rank sum test outcomes for the hypothesis that 3′-UTR lengths of orthologous and non-orthologous genes are the same are P = 2.655e-31 in *M. truncatula* and P = 1.926e-08 in *Arabidopsis*, thereby indicating that the 3′-UTR lengths from orthologous genes are longer than that from non-orthologous genes. **B**. 3′-UTR lengths in pairs of orthologous genes between *M. truncatula* and *Arabidopsis*. Number of orthologous genes is 8876. The Pearson correlation is 0.26 (p-value < 2.2e-16). **C**. 3′-UTR lengths of 2000 randomly-selected and assembled gene pairs between *M. truncatula* and *Arabidopsis*. The Pearson correlation is 0.

In *M. truncatula*, the median length of 3′-UTRs was 180 nts, with the 25^th^ and 75^th^ percentiles being 99 and 255 nts, respectively (Figure 4A, non-orthologous genes). These values were larger than the corresponding lengths in *Arabidopsis* (median = 169 nts, 25^th^-75^th^ percentile range of 129–218 nts; [7]). For both *Arabidopsis* and *M. truncatula*, the 3′-UTR length of genes with orthologs tended to be longer than the length of genes without orthologs (Wilcoxon rank sum test, P = 2.655e-31 in *M. truncatula*; P = 1.926e-08 in *Arabidopsis*). In spite of the differences in 3′-UTR length in *M. truncatula* and *Arabidopsis* genes, there was a weak but significant correlation between 3′-UTR lengths in pairs of orthologous genes between *M. truncatula* and *Arabidopsis* (Figure 4B). In contrast, a plot of 3′-UTR lengths of randomly-selected and assembled gene pairs showed no such correlation (Figure 4C).

**Figure 5:**
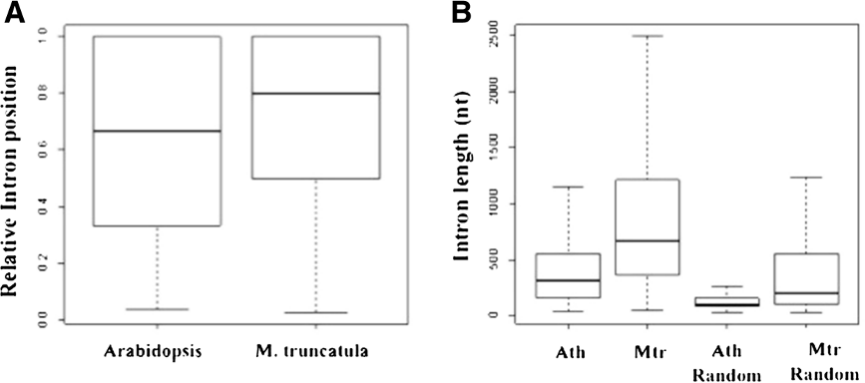
**Comparison of introns with PACs in** ***Arabidopsis*** **and** ***M. truncatula*** **. A**. The relative position of introns with PACs. The y-axis denotes the relative position (in the 5′ to 3′ direction) of the PAC within the intron; y-axis values greater than 0.5 indicate that the respective PAC is nearer the 3′ end than to the 5′ end of the corresponding intron. Numbers of introns are 2296 and 2501 for *Arabidopsis* and *M. truncatula*, respectively. **B**. The lengths of introns with PACs. For the random intron collections, the same numbers of *Arabidopsis* or *M. truncatula* introns were chosen randomly from the complete set of intron sequences. Ath: *Arabidopsis*; Mtr: *M. truncatula*. Median values are 313 nt, 670 nt, 98 nt and 203 nt, respectively.

Of the *M. truncatula* PACs, 2501 mapped to annotated introns (Table 2); these 2501 PACs mapped to 1820 individual introns in 1629 genes. In *Arabidopsis*, about 2300 intron-localized PACs were identified; these PACs occurred in 1841 introns in 1666 genes (Table 2). Introns with PACs tended to occur late in the transcription unit (Figure 5A); additionally, introns with PACs in *M. truncatula* tended to be located closer to the 3′ end than in PAC-containing *Arabidopsis* introns. In both organisms, introns that possess PACs were substantially longer than all introns (Figure 5B).

Amongst the sets of *Arabidopsis* and *M. truncatula* genes with PACs, 10,687 gene pairs consisting of clearly-identifiable orthologs could be identified. Of the 1629 *M. truncatula* genes with intron-situated PACs, 768 had *Arabidopsis* orthologs. However, only 221 of the corresponding *Arabidopsis* orthologs also possessed intron-localized PACs. When 1000 trials were run, each of which consisted of a random selection of 768 genes from the complete set of 10,687 *Arabidopsis* orthologs, an average of 77 were found to possess intronic PACs. Therefore, the coincidence of *M. truncatula* and *Arabidopsis* genes with intronic PACs is significantly different than expected based on the assumption of a random distribution of such genes amongst all genes (χ^2^ test, P = 2.78e-20).

In the set of 221 intronic PAC-containing orthologous gene pairs, only 57 genes were such that both orthologs had an intronic PAC in the same (orthologous) intron. This result indicates that the location of intronic PACs within transcription units is, for the most part, not conserved in the two species.

**Figure 6:**
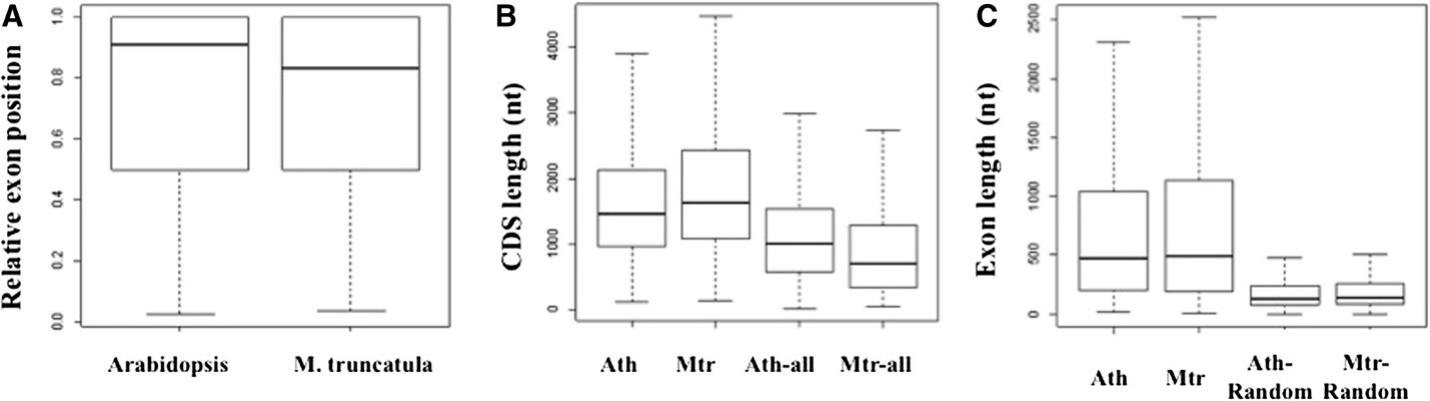
**Comparison of exons or CDS with PACs in** ***Arabidopsis*** **and** ***M. truncatula*** **. A**. The relative position of exons with PACs. The relative position of an exon is the index of the exon divided by the total number of the exons within a gene. A y-axis value >0.5 indicates an exon situated closer to the 3′-end than to the 5′-end of the gene. Numbers of exons are 3759 and 4470 for *Arabidopsis* and *M. truncatula*, respectively. **B**. The lengths of CDS with PACs. The CDS length of all genes in the respective databases was used for comparison. Ath: *Arabidopsis*; Mtr: *M. truncatula* . Median values are 1458 nt, 626 nt, 1008 nt, and 696 nt, respectively. Number of CDS are 2835, 2683, 27406, and 50889, respectively. **C**. The lengths of exons with PACs. For the random exon collections, the same numbers of *Arabidopsis* or *M. truncatula* exons were chosen randomly from the complete set of exon sequences. Ath: *Arabidopsis*; Mtr: *M. truncatula*. Median values are 474 nt, 488 nt, 133 nt and 140 nt, respectively.

Approximately 10% (4470) of the unique *M. truncatula* PACs map to protein-coding regions; these PACs fall within 2683 genes. Exons with these PACs tended to fall nearer to the 3′ ends of their respective genes (Figure 6A). Genes with CDS-localized PACs had slightly longer coding regions than the sets of all *M. truncatula* coding regions (Figure 6B). The slight length difference could be attributed to the observation that individual *M. truncatula* exons that had PACs were substantially longer than the typical exon (Figure 6C). These trends – tendencies of CDS PACs to fall closer to the 3′ end of the CDS, increased lengths of CDS and exons that possess PACs – were also seen in *Arabidopsis* (Figure 6).

For the 2683 *M. truncatula* genes that possess CDS-localized poly(A) sites, 1810 had identifiable *Arabidopsis* orthologs, and 661 of these also possessed CDS-localized PACs. When 1000 trials, each consisting of a random selection of 1810 genes from the complete set of 10,687 *Arabidopsis* orthologs, were performed, an average of 313 genes were found to possess CDS-localized PACs. Therefore, the coincidence of *M. truncatula* and *Arabidopsis* genes with CDS PACs is significantly different than expected based on the assumption of a random distribution of such genes amongst all genes (χ^2^ test, P = 1.15e-38).

2278 (41%) of the 5531 antisense PACs in the *M. truncatula* database could be attributed to convergent transcription; as suggested by Sherstnev et al. [6], their assignment as antisense transcripts are probably computational artifacts and not representative of transcripts that might function as antisense RNAs. However, 3253 (59%) of the antisense PACs in *M. truncatula* are not obviously attributable to overlapping transcription (Cases 3 and 4 in Table 4). These PACs fall within 2397 genes, or 17% of the set of *M. truncatula* genes defined by the complete collection of PACs. Amongst these 2397 genes, 1715 orthologs can be found in *Arabidopsis*. For these 1715 orthologs, 1603 (or 93%) were associated with antisense PACs. In contrast, when 1000 trials were run, each consisting of a random selection of 1715 genes from the complete set of expressed *Arabidopsis* genes, an average of 56% of were associated with antisense PACs. This difference between the random collection and *Arabidopsis* orthologs of *M. truncatula* genes subject to antisense transcription is significant (χ^2^ test, P = 1.98e-138), suggestive of potential significance of these conservation antisense PACs in plants.

## Discussion

### An updated picture of the Medicago truncatula genome

As of version Mt4.0v1 of the *M. truncatula* genome, there are more than 50,000 predicted genes. However, only about 18,000 3′-UTRs are annotated in this version. The data presented in this study permits the addition of some 42,500 poly(A) sites to this annotation, as well as the precise defining of the 3′-UTRs of more than 14,000 *M. truncatula* genes. In addition, these results imply the existence of as many as 5500 as yet unidentified *M. truncatula* genes (these being associated with the PACs that fall within putative intergenic regions). Whether or not most or all of these are protein-coding, or instead if a sizeable fraction encodes non-coding RNAs, is a matter for future study.

In most respects, the characteristics of poly(A) sites and their distributions in *M. truncatula* are similar to those reported for other plants (primarily *Arabidopsis* and rice). Thus, the percentage of *M. truncatula* genes that possess multiple poly(A) sites is considerable (64%) and comparable to the percentages reported for *Arabidopsis* (between 60% and 70%; [5–7]) and rice (between 47% and 82%; [5]). For the most part, the genomic distributions (falling within 3′-UTRs, introns, etc.) of PACs in *M. truncatula* are similar to those seen in *Arabidopsis* (Table 2); the exception to this is the paucity of PACs that map to 5′-UTRs in *M. truncatula.* This probably reflects the relatively incomplete annotation of the *M. truncatula* genome, especially when it comes to the identification of confirmed 5′-UTRs.

As shown in Figure 2, poly(A) sites in *M. truncatula* are associated with the same trends in nucleotide compositions that are seen in other plants [4, 7, 19–21]. Importantly, sites that lie within introns and intergenic regions possess the same signature as sites situated in 3′-UTRs. This indicates that the former sites have a similar tri-partite structure as canonical plant poly(A) sites, a structure that consists of a linear array of (respectively) U-rich region (that may include better-defined submotifs [22, 23]), A-rich element, and U-rich region surrounding the poly(A) site itself [19, 24–26]. These results confirm a general conservation in the poly(A) signal in plants.

As was noted previously in *Arabidopsis* [7], *M. truncatula* poly(A) sites that fall within protein-coding regions have a different sequence profile (Figures 2D, Additional file 2: Figure S1D and S2D). While the nucleotide composition profiles of CDS-localized sites differ substantially from other sites, they are similar to the profile reported for such sites in *Arabidopsis* [7]. The means by which polyadenylation at these sites is accomplished are not known, nor is the significance of this class of poly(A) site understood. However, the conservation in sequence context and in CDS polyadenylation in orthologous genes in *M. truncatula* and *Arabidopsis*, as well as the association of these sites with genes that play roles in stress responses in *Arabidopsis* (Additional file 1: Table S3; also, [7]) are collectively suggestive of distinctive modes of action and function for these sites.

There is a sizeable number of *M. truncatula* PACs that are in an antisense orientation with respect to an annotated gene. A smaller percentage of the *M. truncatula* antisense PACs occur in convergently-transcribed gene pairs than is seen in *Arabidopsis* (41% in *M. truncatula*, vs. 60% in *Arabidopsis* [7]). However, in terms of total numbers of PATs, the overwhelming majority of antisense polyadenylation occurs in convergently-transcribed gene pairs in both organisms (Table 4). This indicates that, as has been noted before for *Arabidopsis* [6, 7], most apparent antisense-oriented poly(A) sites are actually associated with nearby genes, and probably not with dedicated, antisense-oriented non-coding RNAs.

### On the evolution of poly(A) sites in plants

While these generalities demonstrate a broad conservation in the poly(A) signal and thus mechanism of 3′ end formation in plants, they also raise interesting questions. For example, little is known about how poly(A) sites may evolve in orthologous genes in different plant species. Most PACs (and PATs) in both *Arabidopsis* and *M. truncatula* map to established or probable 3′-UTRs (Table 2). The origins of these sites may be via one of two mechanisms – the poly(A) site(s) found in an ancestral gene may be conserved in various lineages, or poly(A) sites may appear and be lost in different lineages. While the latter possibility seems unlikely, the highly degenerate nature of plant poly(A) signals [19, 26] leaves the possibility open. (For example, it has been noted that little more than a general A + U richness may suffice for function as a plant polyadenylation signal [27, 28]. Thus, it is conceivable that modest sequence variation that alters A + U content may be sufficient for the origination of a new polyadenylation signal.) The results presented in this study favor the first model more than the second. Therefore, were poly(A) signals in homologous genes to evolve by random appearance and disappearance, there should be no correlation of the lengths of 3′-UTRs in homologous genes. On the other hand, if an ancestral poly(A) signal is usually retained in various lineages, there should be a correlation in 3′-UTR lengths in homologous genes. The latter is the result that is seen in this study.

Besides 3′-UTRs, poly(A) sites may also occur in other parts of a primary transcript. Intronic poly(A) sites are known to play key roles in *Arabidopsis* (e.g., [29, 30]) and other plants [31–35]. The results of the *M. truncatula* – *Arabidopsis* comparison are curious and contradictory at first glance. On the one hand, there is a modest conservation in terms of the impact of intronic APA on orthologous genes in the two species. However, while being subject to intronic APA is conserved, the position of the intron in affected genes is not. This suggests that the process of intronic APA itself may play roles in gene expression, but that the specific outcomes (which tend to vary for orthologous genes in the two species) are not as important. A similar proposal has been made for alternative splicing outcomes in primates [36]. Thus, although the alternative splicing of transcripts from the DNA polymerase beta locus in primates shows little conservation in terms of specific events, there is nonetheless extensive alternative splicing leading to unproductive transcript isoforms throughout the primate lineage. Moreover, there are indications that these unproductive events have adaptive significance. A similar situation may hold for APA in plants.

While there is a significantly non-random nature to the extent of conservation of intronic APA between *M. truncatula* and *Arabidopsis*, a majority of these events in this study are specific for one or the other species. Thus, as is the case in animals [37, 38], this mode of APA may contribute to a proliferation of species-specific variability in gene expression (through the production of altered mRNA and protein isoforms).

Compared with intronic APA, there is a somewhat greater conservation of CDS-localized poly(A) sites between *Arabidopsis* and *M. truncatula*, as reflected in the greater numbers of orthologous genes that are affected by CDS APA. The association of CDS APA with *Arabidopsis* genes involved in stress responses and post-embryonic development (Additional file 1: Table S3; also, [7]), along with the conservation that is seen in this report, suggests an important role for CDS APA in plants. Because CDS APA removes the translation termination codon from the affected primary transcript, these RNAs and their presumed translation products are likely to be unstable, being rapidly degraded by enzymes associated with non-stop mRNA surveillance [39, 40]. Thus, the most likely function for these mRNAs is a negative one, perhaps serving as part of circuits that fine-tune expression at the mRNA and protein level. The conservation noted in this report suggests that this mode of regulation may be important for gene function.

The bulk of apparent antisense transcription (based on PAT numbers) in *M. truncatula* derives from convergently-transcribed genes, as is the case in *Arabidopsis* [6, 7]. However, 59% of the antisense poly(A) sites (as opposed to individual poly(A) tags) in *M. truncatula* can be dissociated from possible adjacent, convergently-transcribed genes. Interestingly, a high percentage of the associated target genes possess *Arabidopsis* orthologs that are also associated with antisense PACs. This remarkable extent of evolutionary conservation is suggestive of important roles for antisense regulation of these target genes. While such regulation could involve the induction of siRNAs that act to negatively regulate gene expression (e.g., [41]), the observation that there is little correlation between the possible targeting of genes by transcripts defined by antisense PACs and actual target mRNA stability [7] raises the possibility of other modes of regulation. Such modes might include alteration of the chromatin environment surrounding the target. The resolution of these issues awaits further experimental examination.

## Conclusions

To summarize, the results presented here reveal the evolutionary conservation in the plant alternative polyadenylation. They indicate a general conservation in the poly(A) signal in plants, and that the conservation of CDS-localized poly(A) sites in these two species is greater than the conservation of intronic sites. Together, our results suggest some distinct sense and antisense evolution patterns of APA in plants.

## Methods

### Plant materials and PAT-seq library generation

*Medicago truncatula* (Jemalong A17) plants were grown in soil in the greenhouse near the autumn equinox under natural lighting and temperatures between 22-25°C. *M. truncatula* poly(A) tags were generated using total RNA isolated from the combined leaves and washed roots of 3–4 week-old nodule-free plants, following the PAT-seq protocol described in Wu et al. [7]. There is no ethical issue to state for these plants used in the experiment. The production and dataset of the *Arabidopsis* poly(A) tags analyzed here has been described elsewhere [7, 18].

### Sequencing data retrieval and processing

A previous iterative mapping pipeline for paired end sequences was used to determine poly(A) sites [7]. For *Arabidopsis*, the latest genome annotation of TAIR10 was used (ftp://ftp.Arabidopsis.org/home/tair/Genes/TAIR10_genome_release/TAIR10_gff3/TAIR10_GFF3_genes_transposons.gff). The genome sequences and annotation for *M. truncatula* annotation were downloaded from ftp://ftp.jcvi.org/pub/data/m_truncatula/Mt4.0/Assembly/. To prevent double counting of poly(A) sites corresponding to multiple transcripts from the same gene, multiple transcripts from the same gene were merged into one unique gene model. Regions that have different annotations (for example, coding region in one transcript, intron in another, due to alternative splicing) in different transcripts encoded by the same gene were denoted as AMB (AMBiguous).

To facilitate the assignments of PATs and PACs to annotated genes, the annotated genes and 3′-UTRs for *Arabidopsis* and *M. truncatula* were extended using the following reasoning. As described in a previous report [7], for *Arabidopsis*, the annotated 3′-UTRs were extended for 120 nt and genes without annotated 3′-UTR were extended by 338 nt. For *M. truncatula*, about 75% of PACs located in intergenic regions are situated within 400 nt of an adjacent, properly-oriented protein-coding region. In addition, the median 3′-UTR length for *M. truncatula* was found to be 180 nts (Figure 4A). Thus, genes with annotated 3′-UTRs were extended for 200 nt and genes without annotated 3′-UTR were extended by 400 nts; these revised annotations were used to assign PATs and PACs to individual genes.

To study tags that map to more than one gene (Additional file 1: Table S1), tags starting with poly(T) were remapped to the genome using Bowtie [42] with option “-n 1 -l 25 -e 70 -a -m 20”, allowing for multiple occurrences of particular sequences.

### Confirmation of poly(A) sites in *M. truncatula*

EST data was downloaded from the PlantGDB website (http://www.plantgdb.org/download/Download/xGDB/MtGDB/). There are a total of 259,740 ESTs, but only 5497 of them have a poly(A) tail. ESTs with poly(A) tails were extracted and mapped to the *M. truncatula* genome using GMAP [43]. The cDNA-to-genome mapping results were analyzed to get poly(A) sites as described previously [7]. The correspondence of EST- and PAT- derived poly(A) sites was then assessed, making the assumption that EST-derived sites that fall within 50 nts of a given PAC may serve as authentication of the PAC. This was done because of the low numbers of EST-derived poly(A) sites as well as the realization that most plant genes possess more than one site, and that the occurrence of a nearby EST poly(A) site probably reflects the existence of a cluster of nearby sites.

### Analysis of orthologous genes

Orthologous gene pairs between *M. truncatula* and *Arabidopsis* were downloaded from the PLAZA website (ftp://ftp.psb.ugent.be/pub/plaza/plaza_public_02_5/GeneFamilies/genefamily_data.orth.csv.gz). A total of 21,976 orthologous gene pairs were obtained, involving 10275 *Arabidopsis* genes and 6447 *M. truncatula* genes, respectively. 3410 of the *Arabidopsis* genes that are in orthologous groups are such that their *M. truncatula* genes orthologs do not “possess” a PAC.

### Availability of supporting data

The *M. truncatula* dataset supporting the results of this article is available in the NCBI repository with accession number SRA157756 [http://www.ncbi.nlm.nih.gov/sra]. The data compiled and used for the analyses in this article are summarized in the Additional file 4.

## Electronic supplementary material

Additional file 1: Tables S1-S3: This file contains all the Supplemental Tables including the distribution of duplicated PATs and the GO study for *Arabidopsis* genes with CDS PACs. (PDF 1 MB)

Additional file 2: Figures S1 and S2: These figures show the single-nucleotide profiles for PACs defined by single PATs (Additional file 2: Figure S1) and PACs defined by multiple PATs (Additional file 2: Figure S2). (PDF 269 KB)

Additional file 3: **This file contains the** ***M. truncatula*** **annotation with the new poly(A) sites.** The *M. truncatula* PACs are provided in GFF format and the track file is also available for genome browser. (ZIP 952 KB)

Additional file 4: **Spread sheets with summaries of data compiled and used for the analyses in this article.** (XLSX 4 MB)
