# Supplementary material for: Genome-wide determination of poly(A) sites in Medicago truncatula: evolutionary conservation of alternative poly(A) site choice
Source: BMC Genomics. 2014 Jul 21;15(1):615. doi: 10.1186/1471-2164-15-615 (PMC4117952; doi:10.1186/1471-2164-15-615)
Supplement: Supplementary file 1 — Additional file 1: Tables S1-S3: This file contains all the Supplemental Tables including the distribution of duplicated PATs and the GO study for Arabidopsis genes with CDS PACs. (PDF 1 MB) [file 12864_2014_6307_MOESM1_ESM.pdf]

Wu et al., Additional file 1 – Supplemental tables

Table S1. Distribution of PATs that map to multiple positions.

Genomic region	*M. truncatula*				*Arabidopsis*			
	PAT#	PAT%	Dup#	Dup%	PAT#	PAT%	Dup#[3]	Dup%
3'-UTR	196880	24.87%	227841	11.69%	7831	5.97%	13379	5.16%
5'-UTR	109256	13.80%	109267	5.60%	977	0.74%	989	0.38%
AMB	3649	0.46%	4579	0.23%	2779	2.12%	4480	1.73%
CDS	96959	12.25%	163547	8.39%	577	0.44%	921	0.36%
Exon[1]	-	-	-	-	89795	68.44%	177929	68.66%
Intergenic	306657	38.74%	1345189	68.99%	14717	11.22%	33564	12.95%
Promoter[2]	69019	8.72%	84899	4.35%	10962	8.36%	22664	8.75%
Intron	9186	1.16%	14476	0.74%	2665	2.03%	4341	1.68%
Pseudogenic_exon[1]	-	-	-	-	895	0.68%	896	0.35%
Total[4]	791606	100.00%	1949798	100.00%	131198	100.00%	259163	100.00%

[1] The exon and pseudogenic_exon regions are from non-mRNA such as ncRNA and snRNA. For mRNA, the CDS is counted and exon is not. In the *Arabidopsis g*ff file, there are many classes of non-mRNAs. In *M. truncatula*'s GFF file, there are only mRNAs, and no non-mRNA, so no PATs is found in exon or pseudogenic_exon.

[2] The promoter is defined as the 2000 nt region upstream of the 5'-UTR.

[3] "Dup#" is total number of genomic locations that are defined by PATs that map to multiple positions.

[4] Since the numbers in this table are counted within each region, the total number of the unique PATs (PAT#) is not the sum of the PAT# of all the regions.

Table S2. Genomic distributions of PACs with single PAT in *M. truncatula*.

Region[1]	PAC#	PAC%	PAC# **(PAT=1)**	**PAC(PAT=1)/PAC(all)**
3'-UTR	26915	63.2	10574	39.29
5'-UTR	34	0.08	23	67.65
AMB	3104	7.29	1398	45.04
CDS	4470	10.5	2617	58.55
Intergenic	4843	11.3	3032	62.61
Promoter	724	1.70	439	60.64
Intron	2501	5.87	1610	64.37
Total	42591	100	19693	46.24

1: Region designations are as in Table S1.

Table S3. Significant GO terms for *Arabidopsis* genes with CDS PACs [FDR (False Discovery Rate)<0.01].

Ontology[1]	Term	Gene#	FDR
F	nucleoside-triphosphatase activity	51	1.40E-17
F	hydrolase activity, acting on acid anhydrides, in phosphorus-containing anhydrides	51	4.00E-17
F	hydrolase activity, acting on acid anhydrides	51	4.00E-17
F	pyrophosphatase activity	51	4.00E-17
P	post-embryonic development	45	8.50E-15
C	plasma membrane	65	1.30E-14
F	ATPase activity	35	4.00E-14
F	ATPase activity, coupled	29	6.20E-13
F	catalytic activity	210	1.60E-09
P	multicellular organismal process	69	5.50E-09
P	multicellular organismal development	66	1.40E-08
P	developmental process	71	2.60E-08
C	intracellular part	199	3.10E-08
C	intracellular	204	3.80E-08
F	purine ribonucleotide binding	54	4.10E-08
F	ribonucleotide binding	54	4.10E-08
C	intracellular organelle	176	4.60E-08
C	organelle	176	4.60E-08
F	nucleotide binding	67	4.90E-08
C	membrane-bounded organelle	166	5.10E-08
F	ATP binding	48	5.20E-08
P	response to abiotic stimulus	52	5.30E-08
F	adenyl ribonucleotide binding	48	5.60E-08
F	purine nucleotide binding	55	5.80E-08
C	intracellular membrane-bounded organelle	165	6.60E-08
C	cytoplasmic part	141	7.90E-08
C	cytoplasm	150	8.60E-08
F	purine nucleoside binding	49	9.00E-08
F	nucleoside binding	49	9.00E-08
F	adenyl nucleotide binding	49	9.00E-08
P	reproductive developmental process	39	2.50E-07
F	ATPase activity, coupled to transmembrane movement of substances	15	3.50E-07
F	ATPase activity, coupled to movement of substances	15	3.50E-07
F	hydrolase activity, acting on acid anhydrides, catalyzing transmembrane movement of substances	15	3.90E-07
F	glucosyltransferase activity	13	5.30E-07
P	response to stimulus	100	6.80E-07
P	reproductive process	42	7.70E-07
F	hydrolase activity	85	9.40E-07

F	transferase activity	82	1.10E-06
P	reproduction	42	1.20E-06
P	reproductive structure development	37	1.50E-06
F	transferase activity, transferring phosphorus-containing groups	54	1.70E-06
F	ATP-dependent helicase activity	12	1.70E-06
F	kinase activity	49	1.70E-06
F	purine NTP-dependent helicase activity	12	1.70E-06
C	membrane	95	2.10E-06
P	anatomical structure development	52	4.20E-06
P	cellular process	225	6.60E-06
C	nucleus	66	1.00E-05
F	primary active transmembrane transporter activity	15	1.10E-05
F	motor activity	10	1.10E-05
F	helicase activity	13	1.10E-05
F	P-P-bond-hydrolysis-driven transmembrane transporter activity	15	1.10E-05
P	seed development	24	1.60E-05
P	embryonic development ending in seed dormancy	22	2.20E-05
P	cellular nitrogen compound metabolic process	23	2.30E-05
C	cytosol	31	3.00E-05
P	fruit development	24	3.10E-05
P	response to temperature stimulus	22	3.70E-05
F	protein binding	69	3.70E-05
P	response to stress	60	4.40E-05
P	embryonic development	23	4.70E-05
F	phosphotransferase activity, alcohol group as acceptor	35	5.10E-05
P	cellular metabolic process	168	7.60E-05
F	microtubule motor activity	8	9.40E-05
F	cellulose synthase activity	6	0.00012
C	cell part	264	0.00015
C	cell	264	0.00015
P	macromolecule metabolic process	139	0.00023
P	primary metabolic process	169	0.00023
F	transferase activity, transferring hexosyl groups	15	0.00047
P	response to heat	11	0.00048
P	metabolic process	192	0.0005
F	binding	196	0.00058
C	vacuole	16	0.00074
P	developmental growth	13	0.00093
F	inositol or phosphatidylinositol kinase activity	6	0.001
P	response to chemical stimulus	50	0.0015
P	polysaccharide metabolic process	10	0.0015
F	protein kinase activity	27	0.0015
P	multi-organism process	25	0.0016

P	cellular macromolecule metabolic process	122	0.0016
P	regulation of developmental process	16	0.0016
P	growth	15	0.0017
P	response to water deprivation	12	0.0019
P	post-translational protein modification	34	0.0019
P	response to external stimulus	17	0.0019
F	UDP-glucosyltransferase activity	7	0.0023
P	regulation of multicellular organismal process	13	0.0026
P	glucan biosynthetic process	6	0.0026
P	response to water	12	0.0026
F	ATPase activity, coupled to transmembrane movement of ions	6	0.0026
F	transferase activity, transferring glycosyl groups	18	0.0028
C	organelle part	55	0.0028
C	microtubule associated complex	5	0.0028
C	plastid	62	0.0028
C	intracellular organelle part	55	0.0028
P	phosphorylation	30	0.003
F	beta-galactosidase activity	5	0.0035
C	macromolecular complex	48	0.0035
P	polysaccharide biosynthetic process	8	0.0036
P	protein metabolic process	80	0.0037
P	nitrogen compound metabolic process	77	0.0037
P	protein modification process	37	0.0037
P	macromolecule localization	17	0.0037
P	regulation of developmental growth	5	0.0039
P	cellular glucan metabolic process	7	0.0041
F	ATPase activity, coupled to transmembrane movement of ions, phosphorylative mechanism	5	0.0041
P	regulation of biological process	74	0.0044
P	biological regulation	82	0.0044
P	glucan metabolic process	7	0.0044
P	cellular localization	19	0.0044
P	intracellular signaling cascade	21	0.0044
P	cellular amino acid metabolic process	16	0.0044
P	phosphate metabolic process	31	0.0044
P	phosphorus metabolic process	31	0.0044
P	cellular amine metabolic process	16	0.005
P	cellular protein metabolic process	70	0.0056
F	translation factor activity, nucleic acid binding	9	0.0056
F	galactosidase activity	5	0.0057
P	response to wounding	10	0.006
P	organ development	24	0.006
P	response to biotic stimulus	20	0.006
P	RNA metabolic process	39	0.006

P	meristem maintenance	6	0.006
P	system development	24	0.006
P	protein amino acid phosphorylation	26	0.0061
P	microtubule-based movement	5	0.0068
C	chloroplast	56	0.0068
F	oxidoreductase activity, acting on the aldehyde or oxo group of donors, NAD or NADP as acceptor	5	0.0072
C	mitochondrion	31	0.0074
P	oxoacid metabolic process	24	0.0076
P	carboxylic acid metabolic process	24	0.0076
P	cellular carbohydrate metabolic process	15	0.0076
P	organic acid metabolic process	24	0.0076
P	cell surface receptor linked signaling pathway	9	0.008
P	macromolecule modification	38	0.008
P	sulfur amino acid biosynthetic process	5	0.0083
P	regulation of meristem development	6	0.0087
P	establishment of localization in cell	17	0.0097
P	cellular ketone metabolic process	24	0.0099

[1] F, molecular function; P, biological process; C, cellular component.

Note: *Arabidopsis* genes with CDS PACs were selected for gene functional annotation, using the DAVID Bioinformatics Resources (http://david.abcc.ncifcrf.gov/). All *Arabidopsis* genes expressed in this study were selected as background.
