# Supplementary material for: Genome-wide determination of poly(A) sites in Medicago truncatula: evolutionary conservation of alternative poly(A) site choice
Source: BMC Genomics. 2014 Jul 21;15(1):615. doi: 10.1186/1471-2164-15-615 (PMC4117952; doi:10.1186/1471-2164-15-615)
Supplement: Supplementary file 2 — Additional file 2: Figures S1 and S2: These figures show the single-nucleotide profiles for PACs defined by single PATs (Additional file 2: Figure S1) and PACs defined by multiple PATs (Additional file 2: Figure S2). (PDF 269 KB) [file 12864_2014_6307_MOESM2_ESM.pdf]

Wu et al., Additional file 2 – Supplemental figures

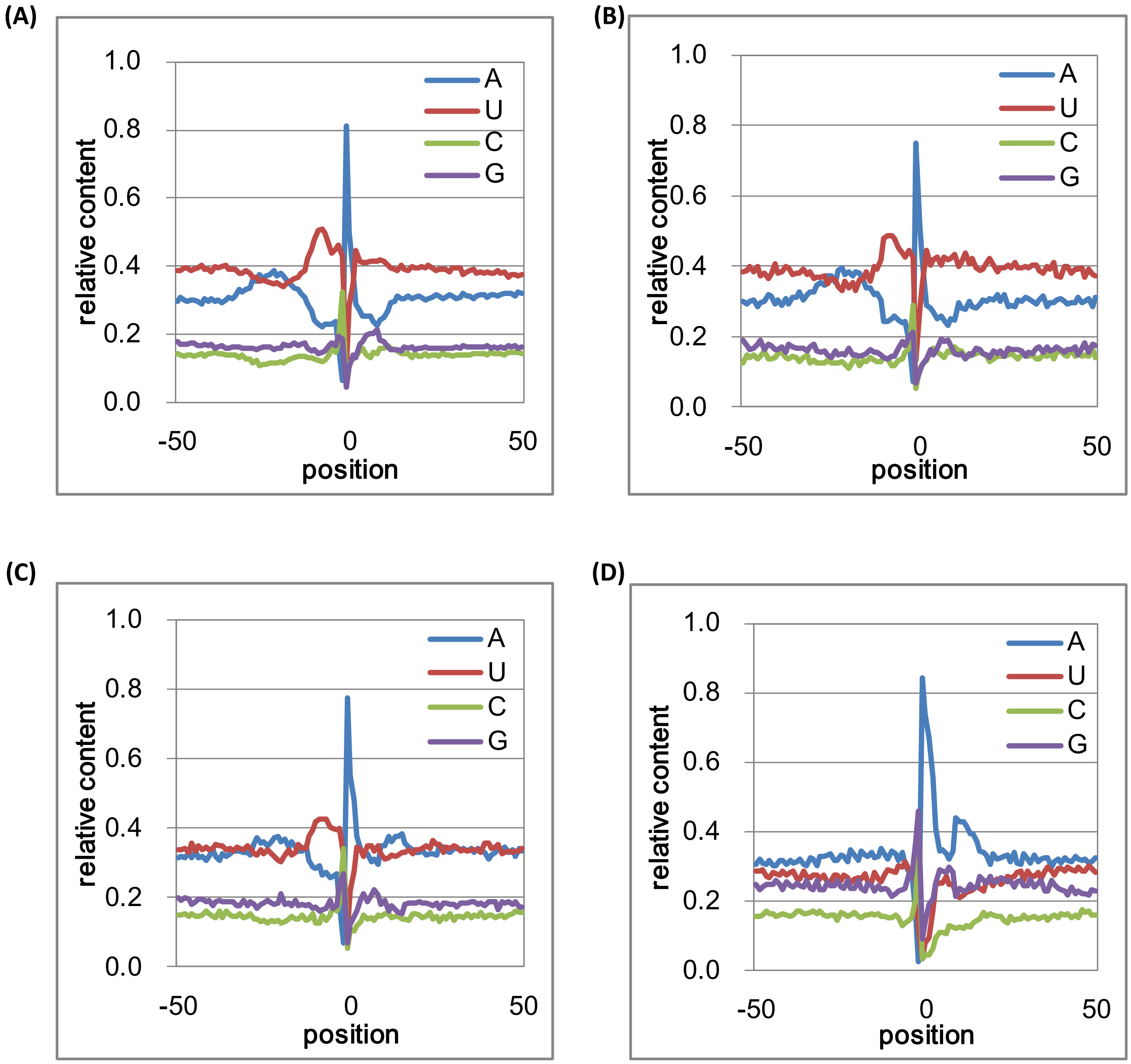

Fig. S1 Nucleotide compositions of the sequences surrounding *M. truncatula* PACs defined by single PAT.
Position-by-position base composition of PACs that map to 3'-UTRs (A), introns (B), intergenic regions (C), protein coding regions (D). Y-axis values are the fractional nucleotide content at each position (plotted along the x-axis); individual traces are color coded as indicated. On the x-axis, "0" denotes the actual cleavage/polyadenylation site.

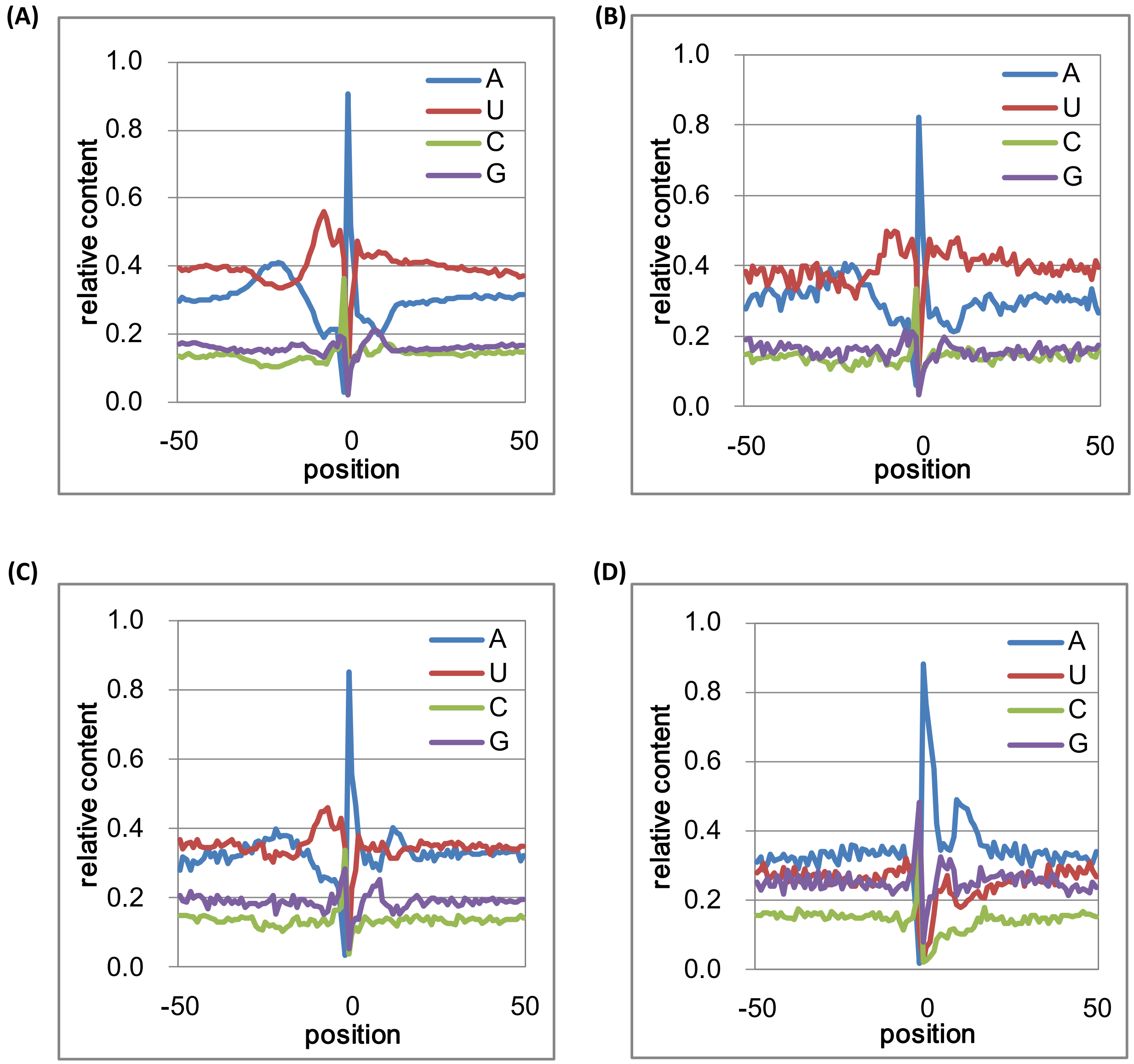

Fig. S2 Nucleotide compositions of the sequences surrounding *M. truncatula* PACs defined by multiple PATs.

Position-by-position base composition of PACs that map to 3'-UTRs (A), introns (B), intergenic regions (C), protein coding regions (D). Y-axis values are the fractional nucleotide content at each position (plotted along the x-axis); individual traces are color coded as indicated. On the x-axis, "0" denotes the actual cleavage/polyadenylation site.
