# Supplementary figures and images for: Genome-wide determination of poly(A) sites in Medicago truncatula: evolutionary conservation of alternative poly(A) site choice
Source: BMC Genomics. 2014 Jul 21;15(1):615. doi: 10.1186/1471-2164-15-615 (PMC4117952; doi:10.1186/1471-2164-15-615)

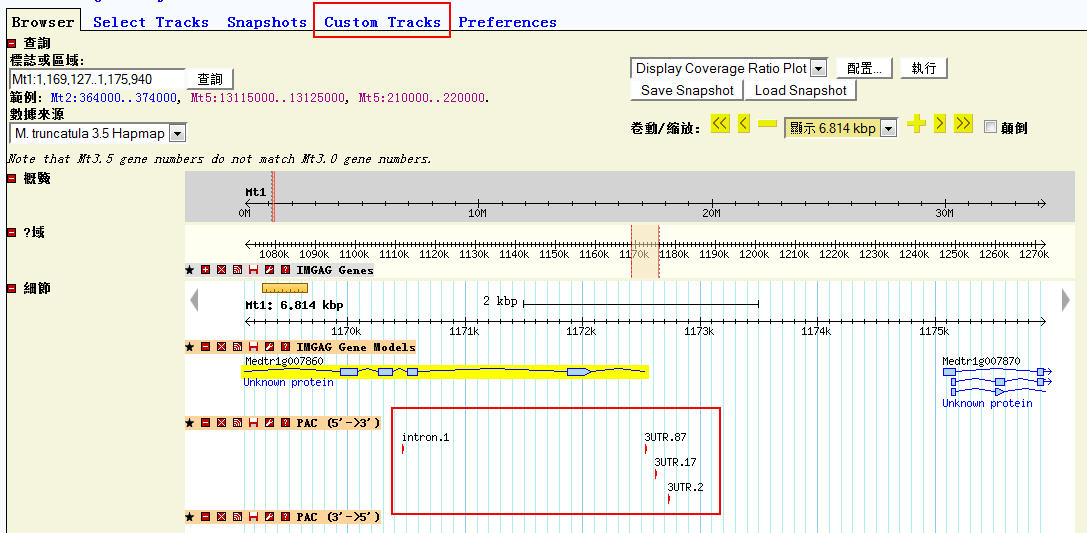

Supplement: Supplementary file 3 — Additional file 3: This file contains the M. truncatula annotation with the new poly(A) sites. The M. truncatula PACs are provided in GFF format and the track file is also available for genome browser. (ZIP 952 KB) [file 12864_2014_6307_MOESM3_ESM.zip › Additional file 1 (Mtr annotation with new PACs).rar Folder/track_example.jpg]
